# Correlation Analysis of the Anterolateral Ligament Length with the Anterior Cruciate Ligament Length and Patient’s Height: An Anatomical Study

**DOI:** 10.1038/s41598-019-46351-0

**Published:** 2019-07-05

**Authors:** Gloria M. Hohenberger, Marco Maier, Angelika M. Schwarz, Peter Grechenig, Andreas H. Weiglein, Georg Hauer, Andreas Leithner, Patrick Sadoghi

**Affiliations:** 1 Department of Orthopedics and Trauma Surgery, Medical University of Graz, Auenbruggerplatz 5, 8036 Graz, Austria; 2 Independent researcher, Vienna, Austria; 3 AUVA-Trauma Hospital Styria I Graz, Göstinger Straße 24, 8020 Graz, Austria; 4 Division of Macroscopic and Clinical Anatomy, Medical University of Graz, Harrachgasse 21, 8010 Graz, Austria

**Keywords:** Ligaments, Skeleton

## Abstract

The aim of this study was to evaluate the anatomical characteristics of the anterolateral ligament of the knee (ALL) with the focus on potential gender differences. The ALL length and the length of the lateral collateral ligament (LCL) were taken in extension. The length of the anterior cruciate ligament (ACL) was measured at 120° flexion. We correlated the length of the ALL with the LCL and ACL with respect to potential gender differences. The ALL was significantly (p = 0.044) shorter in females (mean length: 32.8 mm) compared to males (mean length: 35.7 mm). The length of the ALL correlated significantly positively with the lengths of the ACL (p < 0.001) and the LCL (p < 0.001). There was no significant correlation with the total leg length (TLL) (p = 0.888) and body size (p = 0.046). Furthermore, TLL and donor size correlated significantly positively (p < 0.001). The ALL length correlated significantly positively with the ACL and the LCL length. The ALL length did neither correlate with the TLL nor the donor size. This fact may contribute to planning of graft harvesting in the upcoming techniques for ALL reconstruction.

## Introduction

The anterolateral ligament (ALL) of the knee, also referred to as midthird lateral capsular ligament or anterolateral femorotibial ligament^1^ was primarily described by Segond^2,3^ as a “pearly, resistant, fibrous band” in 1879.

This structure has received increased attention in current anatomical^4–9^ and biomechanical studies^1,10–17^. Various authors have stated that the ALL might contribute to the anterolateral stability of the knee and that undetected injuries of this anatomical characteristic could potentially lead to persistent knee instability following isolated anterior cruciate ligament (ACL) reconstruction^14,18–20^.

However, descriptions regarding the ALL’s anatomical characteristics vary broadly in the literature. Statements regarding its prevalence range from 37.2%^21^ up to 100%^6,7,17,22–28^. Further, disagreements exist whether the ALL is a part of the knee capsule or an extracapsular structure^29^ and its relation to adjacent structures and precise points of origin and insertion remain inconsistent^14–20^. These discrepancies may be traced back to different embalming methods and dissection techniques.

Furthermore, ACL ruptures have been reported more commonly in female athletes^30,31^, however, in comparison little is known about potential gender differences regarding anatomy and pathologies of the ALL.

Therefore, the purpose of our study was to analyse the ALL in a large sample with the main focus on potential gender differences. Further we aimed to correlate the ALL’s length with the lengths of the ACL and the lateral collateral ligament (LCL).

## Material and Methods

### Study sample

The study sample included 104 paired lower extremities gained from human adult cadavers, embalmed using Thiel’s method^32^. Due to obvious signs of interventions, as indicated by local scars, or pathologies, including rupture of the ACL, in the area of interest, 24 extremities were excluded from the study.

### Dissection and measurement pattern

First, the total leg length (TLL), which was defined as the interval between the apex of the greater trochanter and the distal tip of the lateral malleolus, was measured by use of a tape measure.

As the next step, the skin and subcutaneous tissue were removed from the extended knee. The iliotibial tract was incised longitudinally starting 8 cm proximal to the distal tip of the lateral femoral epicondyle to its insertion at Gerdy’s tubercle and dissected to the ventral and dorsal sides. The LCL was palpated with the knee in slight varus. Starting from its proximal portion, the layer encompassing the LCL was incised posterior and parallel to the LCL. In 60° flexion, the fibres forming the ALL were revealed under slight varus.

The relation of the ALL’s proximal portion to the LCL was noted and its total length was taken in extension. The ligament’s width was measured at its femoral and tibial insertions and at the height of the centre of the femoro-tibial joint space. Its thickness was taken at the height of the centre of the femoro-tibial joint space. At the tibial insertion of the ALL, the distances between the anterior and posterior borders of the ligament and the centre of Gerdy’s tubercle and the apex of the fibula were evaluated.

Specimens were inspected regarding fibrous connections between the ALL and the lateral meniscus. Following the ALL’s detachment from the lateral meniscus, the presence of the lateral inferior geniculate artery between these two structures was evaluated.

The LCL’s length was measured in extension and its thickness was evaluated at its broadest part. The length of the anteromedial bundle of the ACL was taken from its femoral attachment at the medial surface of the lateral femoral condyle to the tibial eminence in 120° flexion.

**Figure 1:**
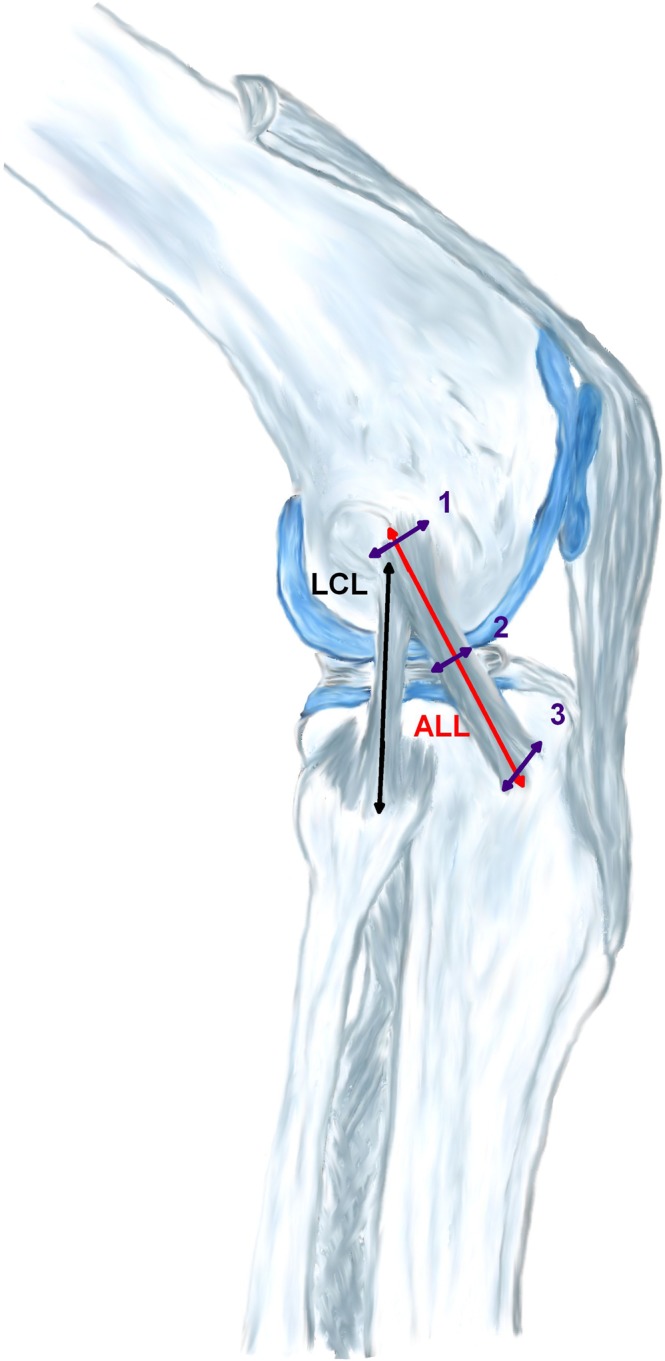
Measurement pattern (Anterolateral ligament [ALL] width at femoral insertion^1^, height of joint space^2^, and tibial insertion^3^).

All measurements were taken with a digital calliper rule (Emil Lux GmbH & Co. KG, Wermelskirchen, Germany; art. No. 572587) and in millimetres by two observers. This device had an accuracy of two decimal places which were rounded to one decimal place. For schematic depiction see Fig. 1.

### Statistical analysis

The collected data were analysed with Spearman’s correlation and *t*-tests to assess associations among variables and differences between males and females using the statistical software R^33,34^. Inter- and intraobserver reliability was calculated for two measurements (time interval between measurements: 10 minutes) of two observers using the *κ*-coefficient, which is a measure of intraobserver agreement for continuous outcomes and ranges from 1 (perfect agreement) to 0 (no agreement). An a priori power analysis was performed. To achieve a statistical a power of 80% at a significance level set to 5% and an estimated difference of 1 cm in length between both sexes, the required number of specimens was n = 80.

Continuous variables are presented as mean and standard deviation (SD), median, minimum and maximum, categorical data as frequencies and percentages.

### Compliance with Ethical Standards

All investigated cadavers were donated to the Division of Macroscopic and Clinical Anatomy of the Medical University Graz under the approval of the Anatomical Donation Program of the Medical University of Graz and according to the Austrian law for donations.

## Results

### Sample characteristics

The mean age of the body donors at time of death was 79.7 years (SD: 10.33; range: 56–95). The mean height was 166.3 cm (SD: 9.3; range: 151–185) for the total collective.

Forty-two knees were gained from female and 38 from male donors. The mean height was 158.2 cm (SD: 3.5; range: 151–165) in the female and 175.2 cm (SD: 3.9; range: 165–185) in the male subgroup.

### Qualitative analysis

**Figure 2:**
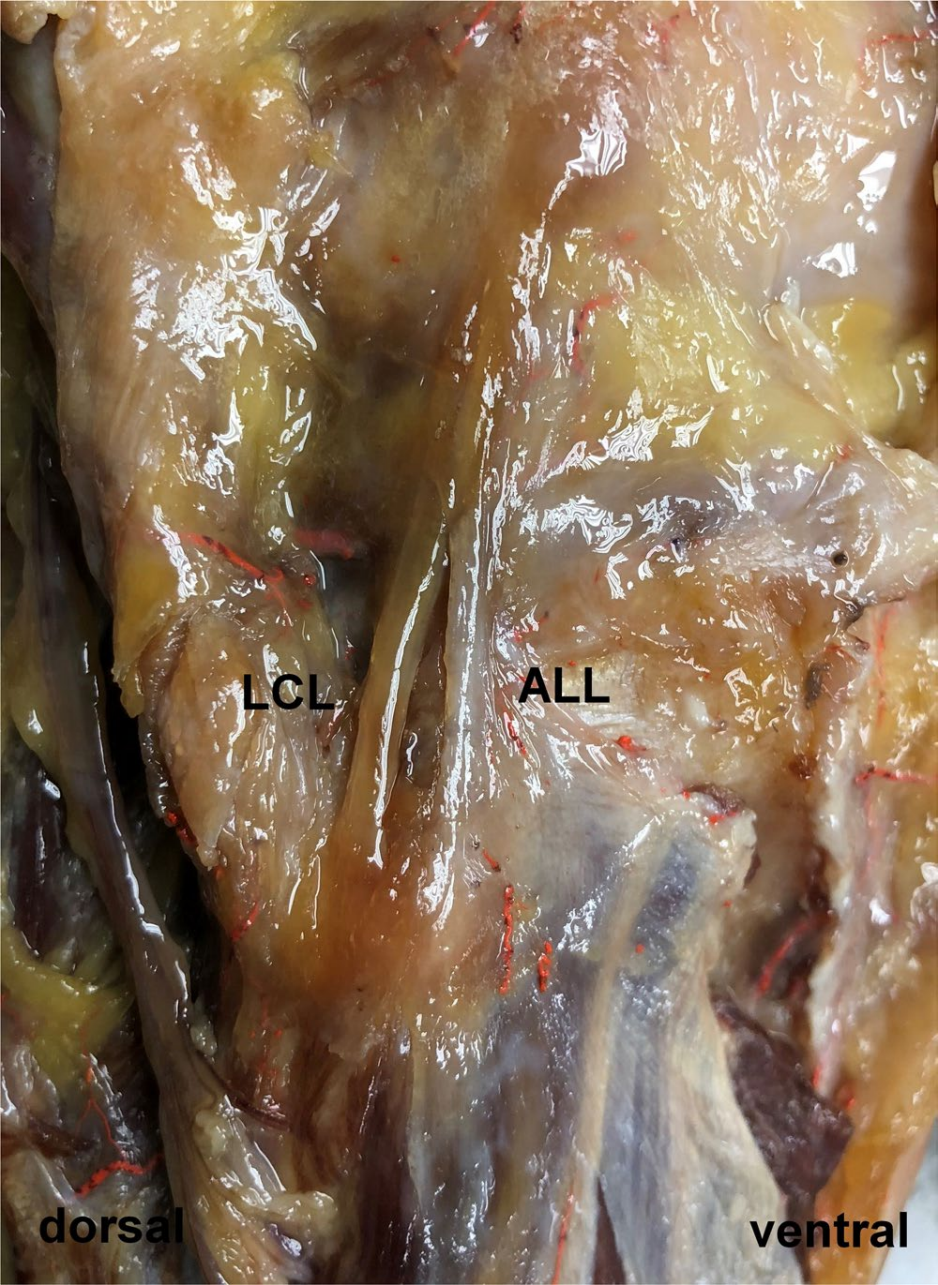
Specimen embalmed by use of Thiel’s method^32^ with dissected lateral collateral ligament (LCL) and anterolateral ligament (ALL).

The ALL could be found in all of the 80 extremities meeting the inclusion criteria as a structure connecting the femur with the tibia (Fig. 2).

In 97.5% of all cases (78/80), the ALL originated from the prominence of the lateral femoral epicondyle anterior to the femoral attachment of the LCL and constantly overlapping the fibres of the LCL. In each one case the ALL’s origin was located remarkably ventral to the LCL without any connection between the two ligaments and in a further specimen proximal to the LCL.

The ligament coursed obliquely to the anterolateral side of the proximal tibia. In 96% (77/80), a connection between the ALL and the lateral meniscus could be observed. This was not present in two cases (3%) and could not be evaluated due to calcifications in one specimen (1%). In 97.5% (78/80), the lateral inferior geniculate artery was found between the lateral meniscus and the ALL after its detachment. In two cases (2.5%), the vessel’s presence could not be evaluated because of calcifications.

### Gender analysis

Detailed descriptive results for the collective are displayed in Tables 1, 2 and 3. On average, male body donors were taller (mean: 175.2 mm; SD: 3.9; range: 165–185) compared to the female collective (mean: 158.2 mm; SD: 3.5; range: 151–165), however the male subgroup was not normally distributed which is why we do not provide statistical significance in this case. The TLL was significantly (t = −5.711, df = 78, p < 0.001) longer in males (mean: 806.2 mm; SD: 45.1; range: 720–897) in comparison to females (mean: 748.3 mm; SD: 44.3; range: 660–818).

**Table 1:** Descriptive characteristics of the total collective (Anterolateral ligament [ALL], fibular head [FH], Gerdy’s tubercle [GT], lateral collateral ligament [LCL], total leg length [TLL]).

n = 80				ALL width							
	Distance GT	Distance FH	ALL length	Femoral insertion	Joint space	Tibial insertion	ALL thickness	TLL	LCL length	LCL thickness	ACL length
mean	16.1	2.6	34.2	8.9	9.3	10.2	2.6	775.8	46.5	4.7	33.6
SD	4.0	3.6	6.3	1.8	1.6	1.5	0.8	53.2	6.9	1.2	4.4
min	7.4	0	22.9	5	5.5	6.6	1.5	660	30.6	1.7	23.4
max	26.7	14.2	53.1	12.5	13	13.3	4.9	897	62	10.5	41.3

**Table 2:** Descriptive characteristics of the female collective (Anterolateral ligament [ALL], fibular head [FH], Gerdy’s tubercle [GT], lateral collateral ligament [LCL], total leg length [TLL]).

n = 42				ALL width							
	Distance GT	Distance FH	ALL length	Femoral insertion	Joint space	Tibial insertion	ALL thickness	TLL	LCL length	LCL thickness	ACL length
mean	14.7	1.8	32.8	8.5	9.1	9.8	2.3	748.3	45.2	4.6	31.7
SD	3.6	3.1	5.2	1.8	1.6	1.5	0.6	44.3	6.7	0.9	4.6
min	7.4	0	22.9	5	5.5	6.6	1.5	660	30.6	2.5	23.4
max	21.9	11	41.3	11.1	12	13	4.1	818	59.2	7	41.3

**Table 3:** Descriptive characteristics of the male donors (Anterolateral ligament [ALL], fibular head [FH], Gerdy’s tubercle [GT], lateral collateral ligament [LCL], total leg length [TLL]).

n = 38				ALL width							
	Distance GT	Distance FH	ALL length	Femoral insertion	Joint space	Tibial insertion	ALL thickness	TLL	LCL length	LCL thickness	ACL length
mean	17.6	3.5	35.7	9.4	9.6	10.7	2.8	806.2	47.9	4.9	35.8
SD	3.9	3.9	7.1	1.7	1.6	1.4	0.9	45.1	6.9	1.5	2.8
min	11	0	24.6	6	5.9	8	1.5	720	35.8	1.7	28.1
max	26.7	14.2	53.1	12.5	13	13.3	4.9	897	62	10.5	41

Further, the ALL was significantly shorter in female (t = −2.048, df = 78, p = 0.044; mean length: 32.8 mm; SD: 5.2; range: 22.9–41.3) than in male body donors (mean length: 35.7 mm; SD: 7.1; range: 24.6–53.1). The ALL was statistically significantly (t = −2.824, df = 64.36, p = 0.006) thinner in females (mean thickness: 2.3 mm; SD: 0.6; range: 1.5–4.1) in comparison to males (mean: 2.8 mm; SD: 0.9; range: 1.5–4.9). The ACL was also significantly longer in males (t = −4.871, df = 68.641, p < 0.001; mean length: 35.8 mm; SD: 2.8; range: 28.1–41) when compared to females (mean: 31.7 mm; SD: 4.6; range: 23.4–41.3). However, there was no significant gender difference concerning the length of the LCL (t = −1.751, df = 78, p = 0.084; females: mean of 45.2 mm; SD: 6.7; range: 30.6–59.2; males: mean of 47.9; SD: 6.9; range: 35.8–62).

### Correlation analysis

The lengths were measured with an almost perfect interobserver (k > 0.8) and intraobserver (k > 0.8) agreement and therefore we showed a high reproducibility of these measurements.

Concerning correlations in the total sample, the ALL correlated significantly positively with the ACL (Spearman’s *ρ* = 0.580, p < 0.001) and the LCL (*ρ* = 0.672, p < 0.001). There was no significant correlation with the TLL (*ρ* = −0.016, p = 0.888) and body size (*ρ* = 0.224, p = 0.046).

The ACL correlated significantly positively with the LCL (*ρ* = 0.423, p < 0.001), the TLL (*ρ* = 0.334, p = 0.002) and the body size (*ρ* = 0.512, p < 0.001). The LCL did neither correlate with the TLL (*ρ* = −0.173, p = 0.124) or donor’s height (*ρ* = 0.132, p = 0.243). Further, TLL and donor size correlated significantly positively (*ρ* = 0.550, p < 0.001).

## Discussion

The aim of our study was to evaluate the ALL with the main focus on potential gender differences. Furthermore, we opted to correlate the ALL’s length with the lengths of the ACL and the LCL.

We found a significantly shorter ALL in females (mean: 32.8 mm; SD: 5.2; range: 22.9–41.3) when compared to males (mean length: 35.7 mm; SD: 7.1; range: 24.6–53.1). The length of the ALL correlated significantly positively with the ACL length (p < 0.001) and the LCL length (p < 0.001). The ALL length did neither correlate with the TLL (p = 0.888) nor the donor size (p = 0.046). The ALL was statistically significantly (p = 0.006) thinner (mean thickness: 2.3 mm; SD: 0.6; range: 1.5–4.1) in females in comparison to male donors (mean: 2.8 mm; SD: 0.9; range: 1.5–4.9).

Although the ALL has been structure of interest in various anatomical, biomechanical, medical imaging and clinical studies, many details concerning its detailed anatomy, function, ideal diagnostic concerning pathologies, indications for therapy and potential treatment options remain unclear.

In the current literature, numbers on prevalence of the ALL are diverging. In Watanabe *et al*.^21^, the ALL was found in solely 37.2% (35/94). Further studies reported a prevalence ranging from 45.5%^8^ to 56.6%^35^. In comparison, higher rates have been stated by Neri *et al*.^36^ with 95% (80/84), by Parker and Smith^37^ including 96.2% (51/53) and Claes *et al*.^5^ who reported a prevalence of 97% (40/41). An ALL-presence of even 100% has been stated by various authors^7,9,22–28^. Our rate of 100% (80/80) is in accordance with the latter studies.

As in Dodds *et al*.^10^ and Runer *et al*.^8^, we found the ALL as an extracapsular structure which was clearly distinguishable from the joint capsule and the surrounding soft tissues.

Results concerning the femoral attachment of the ALL vary. Using the LCL as point of reference, the origin of the ALL has been described as either posterior-proximal^4,35^ or anterior-distal^7,23,38^ to the femoral insertion of the LCL. Additionally, a constant overlapping of the origins of both ligaments has been reported^6,37^. By use of the lateral femoral epicondyle as basing point, the ligament’s insertion has been stated mainly as directly on the prominence of the lateral epicondyle^5,6,37^ or posterior-proximal to it^10,28,36^. As a further variation, the ALL’s femoral origin directly from the popliteus tendon has been described^9,35^. In this study, the femoral attachment of the ALL was found at the prominence of the lateral femoral epicondyle anterior to the femoral attachment of the LCL and constantly overlapping the fibres of the LCL in 97.5% of all cases (78/80). As variations, it was located remarkably ventral to the LCL without any connection between the two ligaments and in a further specimen proximal to the LCL.

Different authors have described the insertion of the ALL as situated approximately hallway between the fibular head and Gerdy’s tubercle^4,5,25,28,35,38^. The possibilities of a closer proximity to either the fibular head^37^ or Gerdy’s tubercle^4^ have been reported. Runer and colleagues^8^ reported mean distances of 15.2 mm from the posterior border of the ALL to the tip of the fibular head and 18.6 mm from the anterior border to the centre of Gerdy’s tubercle. In our sample, the ligament’s posterior border was situated much closer (mean: 2.6 mm) to the fibular head.

Concerning the ALL length in total extension, values from 34.23 mm^37^ up to 44.91 mm^36^ have been reported. In our total collective, the mean length of the ligament was 34.2 mm, which is well comparable to Parker and Smith^37^.

At the femoral attachment, reports about the ALL’s width range from 4.8 mm^4^ to 8.3 mm^5^, whereas the latter value is well comparable to our data (mean: 8.9 mm). Generally, the width at the height of the joint space has been reported as smaller in comparison to the femoral attachment side (Claes *et al*.^5^: 6.7 mm; Runer *et al*.^8^: 5.6 mm; Stijak *et al*.^38^: 4 mm), whereas the ALL broadened in our sample from proximal to distal (mean width at joint space: 9.3 mm). At its tibial insertion, our value (mean: 10.2 mm) is comparable to the literature^4,5,8^.

Regarding the ALL’s thickness, values ranging from 1.2 up to 1.4 mm have been reported^4,5,8,36^. We evaluated a higher value of 2.6 mm in the total collective (females: 2.3 mm, males: 2.8 mm) which is more comparable to Helito *et al*.^7^ (mean thickness of 2.7 mm).

Connections between the ALL and the lateral meniscus haven been described in various studies^4,5,8,10,35,37,39^. Helito *et al*.^40^ found the insertion of the ALL at the lateral meniscus in the transition between the anterior horn and meniscal body in a cadaveric and histological analysis of 33 knees. These data are comparable to Claes *et al*.^5^, who found tight connections between the ALL and the lateral meniscus at the periphery and the middle third of the meniscal body. Claes and colleagues^5^ also found the lateral inferior geniculate artery invariably between the meniscus and the ALL after detachment of the ligament. We found a connection to the lateral meniscus in 96% (77/80) and the lateral inferior geniculate artery in 97.5% (78/80) of all cases.

Concerning imaging studies, Argento *et al*.^41^ were able to identify the ALL via sonography alongside its whole length in 93.8% (150 of 160 cases), respectively 92.5% (148 of 160 cases) by two evaluators. Cavaignac *et al*.^42^ were able to depict the complete ALL in all of their evaluated knees, whereas Capo and colleagues^43^ described the distinction between the ALL and the iliotibial tract as challenging during sonography. Regarding magnetic resonance examinations, Helito *et al*.^22^ depicted the ALL as a whole in 33.3% of all cases, whereas its tibial portion was the part at least encountered. Kosy and colleagues^44^ evaluated 100 magnetic resonance images (MRIs) and were able to visualise the ALL in 94% of all cases including depiction of its meniscal attachment and tibial insertion point in all cases. In Macchi *et al*.^45^, all parts of the ligament could only be observed in 54% of all cases.

The functionality of the ALL has been evaluated in biomechanical trials. Thein *et al*.^46^ tested twelve cadaveric knees with either intact ACLs, sectioned ACL and intact ALL or both the ACL and ALL sectioned by use of a robotic manipulator regarding anterior stability and pivot shift. Authors found that the ALL carries minimal load in the ACL-intact knee during these stability tests. However, in ACL-sectioned knees the load borne by the ALL increased on average to <55% of the load normally borne by the ACL in ligament intact knees. Noyes *et al*.^47^ tested the rotational stability and ACL graft forces in knees with ACL reconstruction and following ALL reconstruction in a cadaveric model. Here, the ALL reconstruction was able to correct small abnormal changes in the internal rotation limit at high flexion angels but it provided only moderate decrease on ACL graft forces and had a minor effect in limiting tibiofemoral compartment translations during pivot-shift tests. Kittl and colleagues^48^ determined the contribution of the anterolateral complex of the knee in 8 ACL-intact and 8 ACL-sectioned knees and found the iliotibial tract as the main restraint for internal rotation, whereas the ALL had a minor function in restraining the pivot-shift. Schon *et al*.^49^ aimed to evaluate the effect of combined ALL and ACL reconstruction and to determine the ideal graft fixation angle for the ALL reconstruction using angles of 0°, 15°, 30°, 45°, 60°, 75°, and 90° in ten fresh-frozen cadaveric specimens. Authors found that combines ACL and ALL reconstruction significantly reduced the rotatory laxity of the knee beyond 30° flexion, however, ALL reconstruction in all tested fixation angles led to overconstraint of the knee. Nitri *et al*.^12^ found in a biomechanical trial that cadaveric knees that had undergone combined ACL- and ALL-repair showed significantly increased rotational stability when compared to knees with isolated ACL-repair and a concomitant ALL lesion.

Tears of the ACL are one of the most common injuries among athletes^49,50^ including ACL reconstruction being the most commonly performed knee ligament surgery^51^ with reported satisfaction rates ranging from 75% to 97%^52^. However, despites advances in reconstruction technologies and the reported satisfactory outcomes, persistence of residual rotational instability following ACL reconstruction has been stated in up to 25% of all cases^53–55^ including 10% to 15% of patients requiring revision surgery^52^. This persisting rotational instability has been discussed to be traced back to an ALL injury^50,52^. Carr *et al*.^52^ aimed to compare the initial prevalence of ALL injuries in patients with ACL reconstruction failure when compared to those without ACL graft failure. However, the incidence of ALL injuries as evaluated on post-trauma MRI scans did not differ between the groups (both groups had eight cases with a completely torn ALL). Claes *et al*.^56^ visualised the ALL in 206 ACL-injured knees. Here, 21.3% of all ALLs were considered uninjured (44/2016) and 78.8% (162/206) showed abnormalities which were most commonly located at the distal part of the ligament (77.8%). Ferretti and colleagues^57^ found ALL injuries in various degrees of severity intraoperatively in 90% (54/60) of a patient sample operated for acute ACL rupture. In all cases, repair of the lesions led to a pivot-shift reduction as intraoperatively tested. Helito *et al*.^58^ evaluated 88 MRIs of patients with acute ACL rupture regarding injuries of the ALL. Hereof, 32.6% (33/88) showed sign of ALL abnormalities which were located in the ligaments’s proximal part in 72% (24/33). Additionally, the meniscal portion of the ALL showed abnormalities in 48% (16/33) of all cases, however no relation was found between meniscal tears and ALL injury. Song *et al*.^59^ evaluated 193 pre-operative MRIs of patients following noncontact ACL trauma concerning the prevalence of bone contusion and concomitant injuries. Authors found that in acute noncontact ACL injuries, the presence of lateral bone contusions is associated with lateral meniscal and ALL abnormalities.

Based on these outcomes, advocacy for ALL reconstruction is recently increasing^49,55,60,61^. Sonnery-Cottet *et al*.^62^ proposed chronic ACL lesion, the presence of a grade 3 pivot shift, participation in high level sports, an associated Segond fracture or a lateral femoral notch sign on conventional radiographs as indications for combined ACL and ALL reconstruction. In technical notes, the use of iliotibial tract^63^ and gracilis autografts^64^ as well as arthroscopic ALL identification^65^ have been described. Sonnery-Cottet and colleagues^20^ re-evaluated the subjective outcomes following combined ACL and ALL reconstruction in 83 patients and reported no specific complications in their sample after a minimum follow-up of two years. Helito *et al*.^60^ compared the results of 33 combined ACL and ALL reconstructions with 68 anatomic intra-articular ACL reconstructions in 101 patients with chronic ACL injury. Authors found significantly better outcomes in the combined reconstruction group regarding International Knee Documentation Committee (IKDC) questionnaire and Lysholm Knee Scoring System evaluation. Lee and colleagues^66^ compared the clinical outcomes between 45 patients undergoing isolated ACL revision surgery with 42 patients with ACL revision in combination with ALL reconstruction. The combined revision group showed significantly reduced rotational laxity and a higher return rate to pre-traumatic sports activities when compared to the singular ACL revision group. However, there was no significant difference regarding anterior laxity between the groups.

We want to outline the following limitations of our work: As our donors are mainly Caucasian (race which includes most natives of Europe, West Asia and North Africa^67^) females and males from Austria, there might be a potential selection bias, so our findings might not be in line with patients from other regions of this world. However, we want to underline the benefit that this is the first study, which analyses this condition in a large series of 80 specimens, which were prepared according to the renowned technique by Thiel^32^.

In conclusion, we found a statistically significantly positive correlation of the ALL length with the ACL and the LCL length. The ALL length did neither correlate with the TLL nor the donor size. This fact may contribute to planning of graft harvesting in the upcoming techniques for ALL reconstruction. Furthermore, female athletes have a 2 to 8 times higher risk for suffering an ACL rupture, which may likely be accompanied by ALL lesions, when compared to males. This increased risk is likely multifactorial including factors as muscle strength, limb alignment, intercondylar notch variations and joint laxity^30^. As an additional factor, a positive correlation between shorter ACLs and injury risk has been described, since shorter ligaments sustain a greater amount of stress during force application on the knee^68^. We found a significantly shorter ALL in females (mean: 32.8 mm) when compared to males (mean: 35.7 mm) and the ALL was significantly thinner in females (mean: 2.3 mm) in comparison to male knees (mean: 2.8 mm). These results represent potential reasons for the increased propensity for ACL tears in female athletes.
